# ID1 and CEBPA coordinate epidermal progenitor cell differentiation

**DOI:** 10.1242/dev.201262

**Published:** 2022-11-16

**Authors:** Christina Geraldine Kantzer, Wei Yang, David Grommisch, Kim Vikhe Patil, Kylie Hin-Man Mak, Vera Shirokova, Maria Genander

**Affiliations:** ^1^ Department of Cell and Molecular Biology, Karolinska Institutet, 171 77, Stockholm, Sweden

**Keywords:** CEBPA, ID1, Differentiation, Epidermal development, Progenitor cell, Transcriptional effectors, Mouse

## Abstract

The regulatory circuits that coordinate epidermal differentiation during development are still not fully understood. Here, we report that the transcriptional regulator ID1 is enriched in mouse basal epidermal progenitor cells and find ID1 expression to be diminished upon differentiation. *In utero* silencing of *Id1* impairs progenitor cell proliferation, leads to precocious delamination of targeted progenitor cells and enables differentiated keratinocytes to retain progenitor markers and characteristics. Transcriptional profiling suggests that ID1 acts by mediating adhesion to the basement membrane while inhibiting spinous layer differentiation. Co-immunoprecipitation reveals ID1 binding to transcriptional regulators of the class I bHLH family. We localize bHLH *Tcf3*, *Tcf4* and *Tcf12* to epidermal progenitor cells during epidermal stratification and establish TCF3 as a downstream effector of ID1-mediated epidermal proliferation. Finally, we identify crosstalk between CEBPA, a known mediator of epidermal differentiation, and *Id1*, and demonstrate that CEBPA antagonizes BMP-induced activation of *Id1*. Our work establishes ID1 as a key coordinator of epidermal development, acting to balance progenitor proliferation with differentiation and unveils how functional crosstalk between CEBPA and *Id1* orchestrates epidermal lineage progression.

## Teaser

**Summary:** ID1 is a key coordinator of epidermal development, acting to balance progenitor proliferation with differentiation. A functional crosstalk between CEBPA and *Id1* orchestrates epidermal lineage progression.

## INTRODUCTION

Progenitor cells in self-renewing tissues commonly undergo a coordinated program of differentiation to accommodate tissue function. Although many aspects of progenitor cell differentiation are known, the transcriptional programs that orchestrate epidermal differentiation during development are not well defined. Epidermal p63^+^ (Trp63) progenitor cells are specified from a single-layered ectoderm at embryonic day (E) 8.5 (Zhao et al., 2015). Following this initial epidermal specification, progenitors commit to stratification and a transient proliferative yet suprabasal cell population co-expressing K5 (Krt5)/K14 (Krt14) progenitor and K10 (Krt10) differentiation marker forms (Koster and Roop, 2007). At around E15, this intermediate cell population initiates terminal differentiation, and proliferation becomes restricted to the basal progenitor layer (Blanpain and Fuchs, 2006). Continued growth of the embryo and expansion of the stratified embryonic epidermis thus requires coordination of progenitor cell cycle exit, delamination and differentiation through transcriptional effectors (Bao et al., 2013; Blanpain et al., 2006; Klein et al., 2017; Mulder et al., 2012; Okuyama et al., 2004). It is likely that seemingly antagonistic transcriptional programs are interdependent (Boxer et al., 2014; Hopkin et al., 2012) and enable fine-tuning of epidermal lineage progression.

Several families of secreted signaling molecules have been implicated in skin development. Epidermal bone morphogenetic protein (BMP) signaling is complex (Blessing et al., 1996), but collective evidence suggests a role in modulating progenitor cell proliferation and promoting differentiation through activation of pSMAD1/5 transcriptional programs (Botchkarev and Sharov, 2004). The inhibitors of differentiation (ID) proteins are established BMP-sensing target genes in several cellular contexts (Genander et al., 2014; Korchynskyi and ten Dijke, 2002; Kowanetz et al., 2004). ID proteins are typically expressed in stem and progenitor cells, only to be downregulated upon differentiation (Lasorella et al., 2001), suggesting – paradoxically from an epidermal BMP signaling perspective – that ID proteins could, as the name implies, enable progenitor self-renewal by suppressing differentiation.

Expression of *Id1* is associated with stemness in several cellular contexts (Jankovic et al., 2007; Liu et al., 2013; Malaguti et al., 2013; Nam and Benezra, 2009; Niola et al., 2012; Romero-Lanman et al., 2012; Ying et al., 2003; Zhang et al., 2014). In the adult skin, ID1 mediates hair follicle stem cell quiescence and is required for hair follicle progenitor cell specification (Genander et al., 2014). In contrast, forced expression of ID1 in immortalized human HaCaT organotypic cultures results in a hyperproliferative, disorganized epidermis (Rotzer et al., 2006) and ID1 is highly expressed in adult human psoriatic skin (Bjorntorp et al., 2003), suggesting that the phenotypic outcome of ID1 is context dependent but impinging on the balance between progenitor cell proliferation and differentiation.

The CEBP family of transcription factors (CCAAT/enhancer binding proteins) are induced upon epidermal differentiation, and couple cell cycle exit with commitment to differentiation in several cell types, including the epidermis (Loomis et al., 2007; Lopez et al., 2009; Oh and Smart, 1998; Zhu et al., 1999). In the hematopoietic lineage, CEBPs are able to redirect chromatin binding of general signaling pathway mediators, including SMAD proteins, thereby acting to define cellular identity during lineage specification and differentiation (Trompouki et al., 2011). In other systems, SMADs bind and inhibit the transcriptional activity of CEBPs (Coyle-Rink et al., 2002; Zauberman et al., 2001), suggesting that crosstalk between SMADs and CEBPs is a commonly employed mechanism in place to fine-tune gene regulation.

Here, we explored published single-cell profiling datasets (Fan et al., 2018) to identify enrichment of *Id1* in E13 progenitor cells committed to differentiation. Employing an *in utero* lentiviral injection strategy (Beronja et al., 2010) to target *in vivo* epidermal progenitors in combination with transcriptional profiling of cultured epidermal progenitor cells allowed us to delineate the consequences of *Id1* silencing on progenitor proliferation and differentiation during the onset of epidermal stratification. We demonstrate that targeting of ID1 leads to a thinning of the developing epidermis, impairs epidermal progenitor proliferation and results in loss of *Id1-*silenced progenitor cells. Furthermore, *in vivo* targeted cells co-express progenitor (K5) and differentiation (K10) markers and transcriptional profiling reveals upregulation of differentiation markers *in vitro*, indicating that ID1 acts to repress epidermal differentiation in progenitor cells.

Co-immunoprecipitation demonstrates that ID1 binds the basic helix-loop-helix (bHLH) TCF family of transcription factors, and we visualize the presence of *Tcf3*, *Tcf4* and *Tcf12* in epidermal progenitor and committed keratinocytes during epidermal stratification. We continue to establish TCF3 as a repressor of epidermal progenitor cell proliferation *in vitro*. Focusing on CCAAT enhancer binding protein alpha (CEBPA) as an ID1-dependent regulator of epidermal cell cycle exit and differentiation *in vivo*, we confirm upregulation of CEBPA after silencing of *Id1* in the epidermis and demonstrate TCF3 binding to *Cebpa* regulatory chromatin. Finally, we unearth a new role for CEBPA in antagonizing BMP-induced *Id1* promoter activation, establishing a regulatory mechanism of ID1 expression in epidermal progenitor cells likely to be relevant for other BMP-sensing genes. Collectively, these data establish ID1 as an essential orchestrator of epidermal differentiation and demonstrate functional crosstalk between CEBPA and ID1, enabling coordinated epidermal progenitor cell differentiation.

## RESULTS

### ID1 is expressed in epidermal progenitor cells during skin development

Although epidermal development has been studied in some detail (Flora and Ezhkova, 2020; Miroshnikova et al., 2019; Miyai et al., 2016; Soares and Zhou, 2018), the process of epidermal differentiation is not fully understood. We exploited a published single-cell RNA-sequencing data set (Fan et al., 2018) from E13 mouse epidermis, a developmental time point where epidermal progenitor cells have not yet initiated terminal differentiation. Re-analysis revealed two main epidermal clusters: cluster 1 was marked by the progenitor cell marker *Krt15*, and differentiation-associated *Krtdap* segregated to cluster 2 (Fig. S1A-C). Gene Ontology (GO) profiling of differentially expressed genes (DEGs) showed enrichment of biological processes linked to tissue and epithelial development in cluster 1, whereas cluster 2 DEGs were associated with epithelial differentiation (Fig. S1D), suggesting that cluster 2 epidermal cells have committed to epidermal differentiation. Interestingly, classification of protein types found in the most significant GO terms in cluster 1 and 2 revealed that epithelial differentiation is associated with DNA-binding transcriptional regulators and cytoskeletal rearrangement (Fig. S1E). Collectively, these results highlight the importance of transcriptional regulation in epidermal commitment to differentiation.

**Fig. 1.**
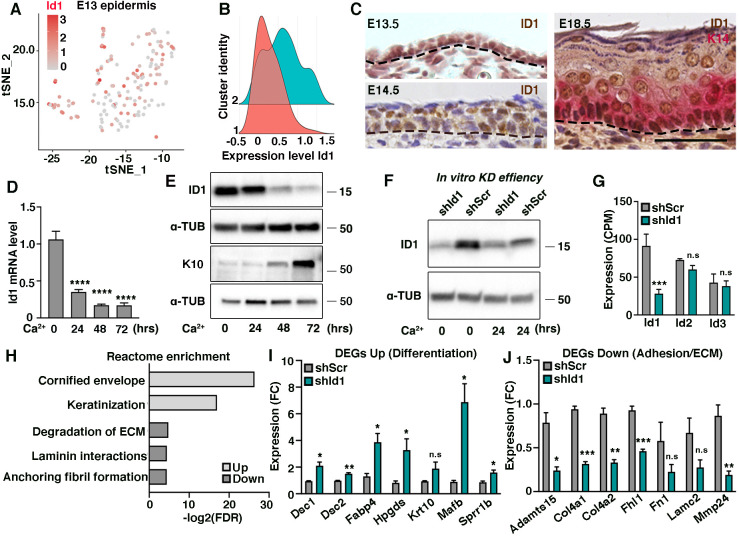
**ID1 is expressed in epidermal progenitor cells during skin development.** (A) Feature plot displaying expression of *Id1* in E13 epidermis. (B) *Id1* expression at E13 epidermis show enrichment of *Id1* in cluster 2 (blue) compared with 1 (red). (C) ID1 protein is abundantly expressed in the E13.5 and E14.5 epidermis. At E18.5, ID1 immunoreactivity is enriched in basal progenitors. (D) *Id1* mRNA expression decreases with differentiation *in vitro*. One-way ANOVA comparing 24, 48 and 72 h with 0 h. (E) ID1 levels are high in progenitor cells and relatively sustained during initiation of *in vitro* differentiation (24 h of Ca^2+^ treatment). K10 is used as a positive differentiation control. (F) Western blot visualizing ID1 protein levels after *shId1* or *shScr* targeting of epidermal progenitors at 0 and 24 h of differentiation. (G) Expression of *Id2* and *Id3* is not altered upon *shId1* targeting compared with *shScr*, displayed as counts per million (CPM) from RNA-sequencing data (*n*=3 in both groups). (H) Reactome enrichment analysis of genes differentially expressed (>2-fold change) in *shId1* compared with *shScr* progenitors. (I,J) Relative expression of selected genes based on reactome enrichment analysis, normalized to expression in *shScr* samples. Dashed line in C,D indicates the boundary between the basal layer of the epidermis and dermis. Data are represented as mean±s.e.m. *P<0.05, **P<0.01, ***P<0.001, ****P<0.0001 using multiple unpaired *t*-test (G,I,J) or ANOVA (D). *n*=3 in quantifications (H-J). n.s, not significant. Scale bar: 50 μm.

In addition to known transcriptional regulators such as *Mafb*, *Hes1* and *Klf4* (Blanpain et al., 2006; Lopez-Pajares et al., 2015; Segre et al., 1999), we identified *Id1* to be enriched in cluster 2 (*P*=0.007, expression fold-change 0.4, likelihood-ratio test, FindAllMarkers in Seurat package), although expressed also in cluster 1 (Fig. 1A,B; Fig. S1A,B,F; Table S1). Localization of ID1 protein in the developing epidermis revealed prominent ID1 expression at E13.5 in most epidermal progenitor cells, in line with the E13 single-cell RNA-sequencing expression data. At E14.5, ID1 immunoreactivity is less uniform and, starting from E15.5, epidermal ID1 expression becomes enriched in basal progenitor cells and is gradually diminished as progenitors delaminate from the basal layer and commit to differentiation (Fig. 1C; Fig. S1G). Analogous to the ID1 *in vivo* expression pattern, cultured primary epidermal progenitors downregulate *Id1* mRNA and corresponding protein when asked to differentiate *in vitro* (Fig. 1D,E). Interestingly, ID1 protein levels are sustained during the first 24 h of differentiation, indicating a role of ID1 in the transition from basal epidermal progenitor to committed epidermal keratinocyte.

In addition to *Id1*, *Id2* and *Id3* are also expressed in the developing epidermis. Expression analysis of *Id2* and *Id3* using E13 single-cell RNA-sequencing fails to reveal enrichment in committed progenitors (cluster 2) (Fig. S1H,I; Table S1). Analysis of Id gene co-expression shows that more than half (59%) of E13 epidermal progenitors express *Id1* but not *Id2* or *Id3*, whereas 36% of progenitor cells co-express all three Id genes. Only a small percentage (5%) of progenitors at E13 are Id gene negative (Fig. S1J). These data suggest that the family of ID proteins likely have both redundant and non-redundant functions in epidermal development. Based on the statistically significant enrichment of *Id1*, but not *Id2* or *Id3*, in cluster 2 (Table S1), we focus on delineating the role of ID1 in the developing epidermis.

### ID1 is associated with epidermal lineage progression

To address the function of ID1 in epidermal progenitor cells, we transduced primary epidermal progenitor cells with either control scrambled shRNA (*shScr*) or an shRNA-targeting *Id1* (*shId1*) (Fig. 1F). In line with the distinct expression profiles of *Id1* compared with *Id2* and *Id3* (Fig. S1H,I), silencing of *Id1* in cultured epidermal progenitors did not lead to a compensatory upregulation of *Id2* and *Id3* mRNA (Fig. 1G). Reactome pathway analysis including DEGs upregulated in *shId1* compared with *shScr*-transduced primary epidermal progenitors revealed biological processes associated with formation of the cornified envelope and keratinization, indicative of epidermal differentiation (reactome enrichment *P*=7*10^−12^ and 9*10^−9^, respectively). In contrast, downregulated DEGs were linked to extracellular matrix (ECM) modulation and laminin signaling (reactome enrichment *P*=2.8*10^−5^, *P*=1.4*10^−4^ and *P*=8.7*10^−5^) (Fig. 1H-J; Fig. S1K), suggesting that *Id1* influences gene programs altering the composition of the ECM and/or adhesion to the basement membrane. All in all, the *in vivo* ID1 expression pattern, in combination with transcriptional profiling, associates ID1 with epidermal lineage progression.

### ID1 counteracts epidermal progenitor delamination

**Fig. 2.**
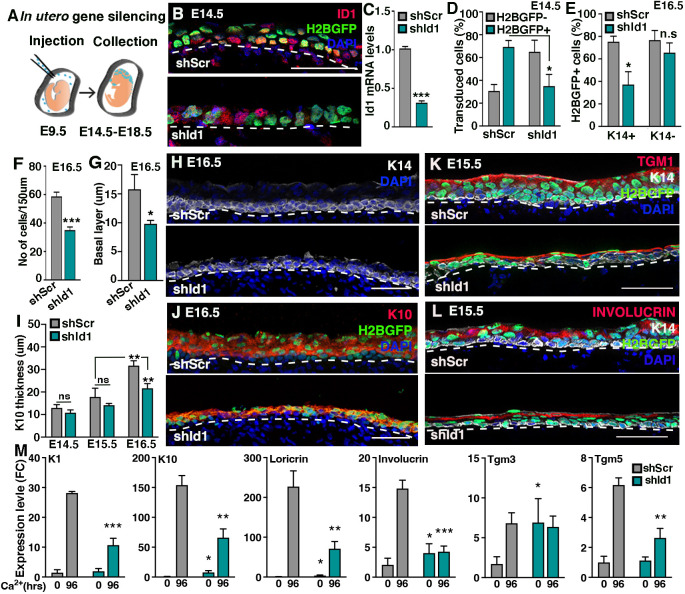
**ID1 counteracts epidermal progenitor delamination.** (A) Schematic of *in utero* lentiviral injections. (B) E14.5 ID1 immunoreactivity is reduced in H2BGFP-positive cells upon injection of lentiviral *shId1*, but not control *shScr*. (C) *Id1* mRNA expression is reduced in epidermal progenitors targeted *in vitro* with *shId1* compared with *shScr*. (D) The percentage of transduced H2BGFP-positive epidermal progenitors is reduced in *shId1*-targeted epidermis compared with *shScr* at E14.5. (E) Quantification of percentage of H2BGFP-positive targeted K14-expressing basal progenitors and H2BGFP-positive K14 negative suprabasal keratinocytes indicate selective loss of *shId1*-targeted progenitor cells. (F) Total number of epidermal nuclei per 150 μm is significantly reduced in *shId1* compared with *shScr*-targeted epidermis at E16.5. (G) Quantification of K14-positive basal progenitor layer thickness in *shId1* and *shScr* epidermis at E16.5. (H) Representative images of K14 thickness at E16.5 in *shId1* and *shScr* epidermis. (I) K10-positive spinous layer thickens with epidermal development. Silencing of *Id1* impairs spinous layer development. (J) Representative images of K10 distribution at E16.5 in *shId1* and *shScr* epidermis. (K,L) Terminal differentiation is temporally and spatially normal in *shId1* compared with *shScr* as judged by expression of differentiation marker TGM1 and involucrin at E15.5. (M) Differentiation markers expression in *shScr* and *shId1* cells. Statistical analysis is performed between *shScr* and *shId1* at 0 or 96 h. Dashed line in B,H,J-L indicates the boundary between the basal layer of the epidermis and dermis. Data are represented as mean±s.d. *P<0.05, **P<0.01, ***P<0.001 using multiple unpaired *t*-test. *n*=3-8 in quantifications (D-F,I,M), *n*=2-3 in G. Scale bars: 50 μm.

To assess the function of ID1 *in vivo*, we exploited the method of ultrasound-guided *in utero* injections of high titer lentiviral particles (Beronja et al., 2010) (Fig. 2A). *Id1* was either silenced in wild-type mice using an shRNA-targeting *Id1*, or depleted by injection of a CRE-expressing lentivirus (LV-CRE) into a conditional *Id1* transgenic (*Id1^fl/fl^*) mouse line at E9.5 (Nam and Benezra, 2009). Delivery of *shId1* (as judged by H2BGFP reporter expression) successfully reduced *in vivo* ID1 protein expression at E14.5 when compared with embryos targeted with *shScr*, in line with *in vitro* knockdown efficiency (Fig. 2B,C). Similarly, LV-CRE injection resulted in efficient loss of ID1 immunoreactivity in *Id1^fl/fl^*-targeted epidermal progenitors at E14.5, whereas ID1 protein was sustained in *Id1^+/fl^*-targeted skin (Fig. S2A), indicating that injection of either *shId1* or LV-CRE efficiently ablated epidermal ID1 expression *in vivo*.

Even though the transduction efficiency of *shId1* and *shScr* were comparable when cultured epidermal progenitors were infected (Fig. S2B), the percentage of H2BGFP reporter-expressing progenitors was reduced in E14.5 *shId1* epidermis compared with *shScr* (Fig. 2D; Fig. S2C). At E16.5, *shId1* embryos displayed significantly fewer H2BGFP/K14-positive progenitor cells compared with embryos targeted with *shScr*, whereas the number of H2BGFP-positive, K14-negative suprabasal cells remained high (Fig. 2E; Fig. S2D). At E18.5, *shId1*-targeted basal progenitor cells were scarce and remaining epidermal H2BGFP expression was localized to K14-negative suprabasal keratinocytes or developing hair follicles (Fig. S2E). Analogously, we found a reduction of the number of *Id1*-silenced progenitor cells at E16.5 in *Id1^fl/fl^* embryos compared with E14.5 (Fig. S2F-I), indicating that progenitors lacking ID1 are selectively lost during epidermal development.

To assess whether loss of *Id1*-targeted progenitors is compensated by untargeted progenitors, we quantified the total number of cells in *shId1* epidermis at E14.5 and E16.5. We found a reduction in the epidermal cell number at E16.5, but not E14.5, in *shId1* epidermis compared with *shScr*-targeted embryos (Fig. 2F; Fig. S2J). We then analyzed apoptosis to see whether cell death could explain the selective loss of *Id1*-depleted progenitors, but did not detect increased CC3-immunoreactivity in *shId1* compared with *shScr*-targeted E14.5 epidermis (Fig. S2K,L). Collectively, these results suggest that ID1 counteracts precocious epidermal progenitor cell delamination, independent of apoptosis.

### Loss of ID1 results in epidermal thinning without affecting terminal differentiation

To assess whether the reduction of epidermal cell number at E16.5 corresponded to loss of progenitor or differentiated cell layers, we first quantified the thickness of the entire epidermis as well as the basal K14-positive progenitor layer and found both to be diminished when comparing *shId1* with *shScr* embryos (Fig. 2G,H; Fig. S2M). As our *in vitro* transcriptional profiling implicated ID1 in epidermal differentiation (Fig. 1I,J), we characterized the dynamic formation of the K10-expressing spinous layer between E14.5 and E16.5. Whereas the thickness of the spinous layer increased in *shScr*-targeted epidermis during development, it failed to do so efficiently in *shId1*-infected epidermis (Fig. 2I), and at E16.5 there was a significant reduction in the thickness of the spinous layer in *shId1* epidermis compared with *shScr*-targeted epidermis (Fig. 2I,J). Owing to the rapid loss and few remaining CRE-targeted cells in *Id1^fl/fl^* epidermis (Fig. S2H), we failed to detect any reduction in either the E16.5 spinous layer or the overall epidermal thickness (Fig. S2N-P), suggesting that the expanding epidermis is able to compensate for the mosaic genetic depletion of ID1.

*In vivo* ID1 expression pattern is consistent with a role for ID1 in commitment to differentiation rather than terminal differentiation. To assess whether the absence of ID1 induced precocious terminal differentiation *in vivo*, we analyzed E15.5 epidermis, the earliest time point at which we could confidently detect markers of terminal differentiation. Although we found a general thinning of the spinous and granular differentiated cell layers marked by TGM1 and involucrin in *shId1* epidermis compared with *shScr*-transduced skin (Fig. 2K,L), TGM1 and involucrin were correctly expressed, temporally as well as spatially. Cultured *shId1* and *shScr* progenitor cells both upregulated spinous and granular differentiation markers when asked to terminally differentiate for 96 h *in vitro* (Fig. 2M), although the upregulation was less prominent when *Id1* was silenced. In line with our transcriptional profiling (Fig. 1I,J), differentiation markers [with the exception of *K1* (*Krt1*) and *Tgm5*] were enriched in *shId1* compared with *shScr* progenitor cells before differentiation. Taken together, these data suggest that progenitor cells lacking ID1 do not initiate terminal differentiation precociously.

### Progenitor cells devoid of ID1 co-express basal and differentiation markers

**Fig. 3.**
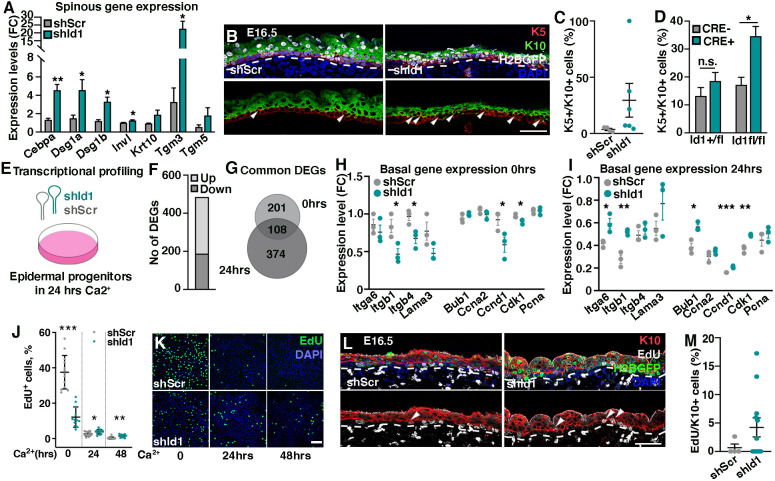
**Progenitor cells devoid of ID1 co-express basal and differentiation markers.** (A) Spinous marker expression after silencing of *Id1* in cultured epidermal progenitor cells. (B,C) *In vivo* silencing of *Id1* increases the incidence of K5/K10 double-positive cells at E16.5 compared with *shScr*-targeted epidermis. Arrowheads indicate K5/K10 double-positive cells. Dashed line indicates the boundary between the basal layer of the epidermis and dermis. (D) The percentage of K5/K10 double-positive cells is significantly increased in *Id1^fl/fl^* compared with *Id1^+/fl^* epidermis when ID1 is ablated using a CRE lentivirus. (E) Cultured epidermal progenitors were targeted with *shId1* or *shScr* and asked to differentiate for 24 h before collection and transcriptional profiling. (F) Number of differentially expressed genes (DEGs) in *shId1* compared to *shScr* targeted cells at 24 h of differentiation. (G) Overlap between differentially expressed genes at 0 and 24 h of differentiation. (H) Genes associated with a basal progenitor cell state are reduced in *shId1* compared with *shScr*-targeted epidermal progenitor cells (*n*=3). (I) Basal genes are sustained in *shId1*-targeted progenitors upon differentiation. (J,K) EdU incorporation upon differentiation of *shId1*- and *shScr*-infected progenitors (*n*≥12 for each sample). (L,M) *In vivo* silencing of *Id1* leads to an increased number of suprabasal cells co-expressing EdU and K10. Arrowheads indicate EdU/K10 double-positive cells. Dashed line indicates the boundary between the basal layer of the epidermis and dermis. Data are represented as mean±s.e.m. *P<0.05, **P<0.01, ***P<0.001 using multiple unpaired *t*-test. *n*=3-6 in quantifications (A,C,D,H,I,M). n.s, not significant. Scale bars: 50 μm (B,L); 100 μm (K).

During epidermal development, basal progenitor cell crowding induces delamination and subsequent differentiation (Miroshnikova et al., 2018). Delaminating epidermal progenitors couple induction of spinous fate markers with suppression of basal gene programs (Blanpain et al., 2006). Revisiting our transcriptomics analysis (Fig. 1H), we identified upregulation of spinous markers in *shId1*-targeted epidermal progenitors *in vitro* (Fig. 3A), suggesting that the basal-to-spinous transition is affected in the absence of ID1. Analysis of basal (K5) and spinous (K10) marker co-expression in E16.5 epidermis revealed enrichment of K5/K10 double-positive epidermal progenitors in *shId1* compared with *shScr* (Fig. 3B,C). Analogously, the percentage of K5/K10 double-positive progenitors was significantly increased in LV-CRE-targeted *Id1^fl/fl^* compared with *Id1^+/fl^* epidermis (Fig. 3D; Fig. S3A), indicating that loss of ID1 leads to an accumulation of cells co-expressing markers normally representative of distinct cell states.

### ID1 co-ordinates the basal-to-suprabasal progenitor state transition

Interestingly, most cells with double progenitor/differentiation marker expression were not in contact with the basement membrane (Fig. 3B), suggesting that ID1-silenced progenitors failed to downregulate progenitor markers during delamination. To this aim, we sequenced *shId1*- and *shScr*-targeted epidermal progenitors after 24 h of *in vitro* differentiation (Fig. 3E,F). Reactome analysis confirmed the involvement of ID1 in coupling inhibition of differentiation to modulation of ECM (Fig. S3B). Interestingly, 35% of the DEGs found in *shId1*-targeted epidermal progenitors were significantly altered upon differentiation of *shId1* progenitors (Fig. 3G), suggesting that aspects of ID1 function are conserved in basal progenitors and differentiating keratinocytes.

Focusing on markers associated with the basal state revealed that silencing of *Id1* in progenitor cells resulted in reduced expression of genes linked to basal membrane anchoring and cell cycle progression (Fig. 3H), potentially coupling loss of ID1 to progenitor cell delamination and cell cycle exit. Paradoxically however, comparing transcriptional profiles of *shId1* and *shScr* progenitors after 24 h of differentiation revealed that *shId1*-targeted progenitors sustained expression of basal markers normally lost during differentiation (Fig. 3I). These data suggest that, although epidermal progenitors downregulate basal markers upon ID1 silencing, their response to *in vitro* differentiation cues is impaired.

To address whether silencing of *Id1* not only affects expression of a cohort of basal genes, but also affects basal cell characteristics during lineage progression, we pulsed *in vitro* differentiated epidermal progenitors with EdU. As expected, control *shScr* progenitors rapidly exited the cell cycle and were largely EdU-negative at 24 h of differentiation; however, a significant fraction of differentiated progenitors targeted with *shId1* still incorporated EdU after 48 h of differentiation (Fig. 3J,K). In addition, we observed an enrichment of suprabasal EdU/K10 double-positive progenitors in *shId1*-silenced epidermis normally absent in *shScr*-targeted epidermis at E16.5 (Fig. 3L,M). Our work suggests that ID1 impinges on the basal-to-suprabasal transition, acting to co-ordinate and fine-tune progenitor as well as differentiation gene programs throughout epidermal lineage transition.

### Epidermal progenitor proliferation is positively regulated by ID1

**Fig. 4.**
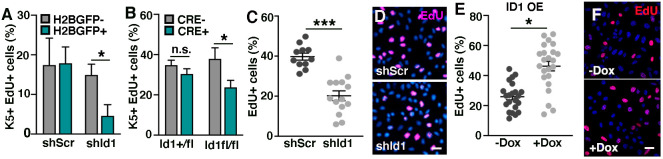
**Epidermal progenitor proliferation is positively regulated by ID1.** (A) Percentage of *in vivo* K5/EdU-positive proliferating progenitors are reduced upon *shId1* targeting compared with *shScr*-targeted epidermis at E16.5. (B) LV-CRE-mediated *Id1* ablation reduces proliferation in K5-positive epidermal progenitors in *Id1^fl/fl^* compared with *Id1^+/fl^* epidermis. (C,D) Silencing of *Id1* in cultured epidermal progenitor cells leads to reduced EdU incorporation. (E,F) Overexpression of ID1 in cultured epidermal progenitors leads to increased EdU incorporation. Data are represented as mean±s.e.m. *P<0.05, ***P<0.001 using multiple unpaired *t*-test. *n*=3 in A,B. Scale bars: 25 μm.

Tissue development requires regulation of mechanisms balancing progenitor cell proliferation and differentiation. As downregulation of ID1 leads to diminished expression of basal progenitor cell markers (Fig. 3H), including the cell cycle regulator *Ccnd1*, we analyzed epidermal progenitor proliferation after manipulation of *Id1*. *In vivo* targeting of *Id1* reduced progenitor cell proliferation using either an shRNA or LV-CRE strategy (Fig. 4A,B; Fig. S4A), and cultured primary *shId1* epidermal progenitor cells incorporated significantly less EdU than *shScr* progenitors (Fig. 4C,D). In contrast, overexpression of ID1 in primary epidermal progenitor cells increased EdU incorporation *in vitro* (Fig. 4E,F; Fig. S4B,C), suggesting that ID1 positively regulates progenitor renewal during epidermal development.

### Identification of ID1 gene signatures

**Fig. 5.**
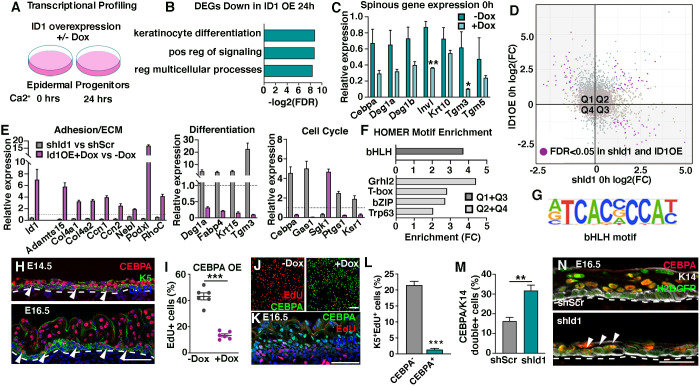
**Identification of ID1 gene signatures.** (A) ID1 is induced in a doxycycline-dependent manner and progenitor cells are differentiated and profiled (*n*=3). (B) Downregulated genes after ID1 overexpression and 24 h of differentiation. DEGs, differentially expressed genes. (C) Spinous gene expression is reduced in epidermal progenitors upon ID1 overexpression. (D) Identification of ID1 gene signatures by merging expression data from *shId1* and ID1 overexpression profiling of epidermal progenitors reveals a cohort of statistically significant genes (marked in purple) (LogFC >1 and FDR >0.05). Q1 and Q3 represent genes for which expression correlates to modulation of ID1. Q2 and Q4 represent genes that are only induced (Q2) or repressed (Q4) when ID1 levels are altered. (E) ID1 signature genes are functionally linked to adhesion and extracellular matrix (ECM) modulation, differentiation or cell cycle regulation (derived from Q1/Q3). (F) HOMER transcription factor binding motif analysis show distinct enrichment binding motifs in promoters (+400 bp from transcription start) of genes found in Q1+Q3 compared with promoters in the expressed transcriptome. (G) bHLH motif enriched in Q1+Q3 gene promoters. (H) Immunoreactivity of CEBPA and K5 in E14.5 and E16.5 localizes CEBPA to suprabasal keratinocytes and scattered basal K5-positive progenitors. Arrowheads indicate CEBPA-positive basal cells. Dashed line indicates the boundary between the basal layer of the epidermis and dermis. (I,J) Overexpression of CEBPA significantly reduces EdU incorporation *in vitro*. (K,L) Proliferating K5-positive progenitors are largely CEBPA negative. (M,N) K14/CEBPA double-positive progenitors are significantly enriched in *shId1*-targeted epidermis when compared with *shScr*. Arrowheads highlight K14/CEBPA positive cells. Dashed line indicates the boundary between the basal layer of the epidermis and dermis. Data are represented as mean±s.e.m. (C,E,I,M) and mean±s.d. (L). *P<0.05, **P<0.01, ***P<0.001 using multiple unpaired *t*-test. *n*=3-6 for quantifications (C,E,L,M). Scale bars: 50 μm (H,K,N); 100 μm (J).

Our data positions ID1 at the intersection of proliferation and differentiation in epidermal progenitor cells. Reasoning that silencing of *Id1* negatively affects proliferation and leads to an induction of differentiation markers, forced ID1 expression would, in addition to driving cell cycle progression, shift gene expression profiles towards a basal state. To this end, we profiled epidermal progenitor cells overexpressing ID1, as well as ID1-overexpressing progenitors asked to differentiate for 24 h (Fig. 5A; Fig. S5A). Focusing on downregulated DEGs (>2-fold change) at 24 h of differentiation, GO analysis revealed significantly enriched biological processes such as epidermal progenitor cell differentiation and regulation of signaling (Fig. 5B), suggesting that differentiation is hampered when ID1 is overexpressed. Expression levels of spinous markers elevated in *shId1* progenitors were found to be reduced in ID1-overexpressing progenitors compared with uninduced progenitors, albeit from a low starting level (Fig. 5C). After 24 h of differentiation, induction of spinous gene expression in ID1-overexpressing cells was prominent, although hampered, when compared with uninduced differentiated cells (Fig. S5B). In contrast to cells silenced for ID1, we did not find alterations in basal gene expression (Fig. S5C,D). These data suggest that sustained ID1 expression during differentiation acts to slow down, rather than inhibit, epidermal differentiation.

Combining transcriptional profiling results from both *shId1* and ID1 overexpression in epidermal progenitor cells allowed us to define a set of 83 candidate *Id1* target genes, the expression changes of which correlated significantly (FDR>0.05) with alteration of *Id1* transcript levels (Fig. 5D, Q1 and Q3; Table S2). Within this relatively small group of candidates, we identified cohorts of genes representing aspects of the *in vivo* phenotypes described upon *Id1* silencing (Fig. 5E), correlating alterations in *Id1*-associated gene signatures with ECM modulation, differentiation and cell cycle regulation.

### ID1 interacts directly with bHLH transcription factors

Lacking a DNA binding domain, ID1 is unable to affect transcription through direct chromatin interaction at target genes (Massari and Murre, 2000). To mechanistically begin to understand how ID1 affects epidermal progenitor cells, we aimed to identify epidermal ID1 binding partners. ID1, as well as family members ID2 and ID3, were overexpressed in cultured epidermal progenitor cells, after which ID-binding protein complexes were isolated and analyzed using mass spectrometry (Fig. S5E,F). We identified three known class I bHLH transcription factors, TCF3 (E2A), TCF4 (ITF2) and TCF12 (HEB), to be bound to ID1, whereas ID2 and ID3 associated exclusively with TCF12 (Table S3). Other published ID1-interacting transcription factors (Roberts et al., 2001; Yates et al., 1999) were not identified, suggesting that ID1 predominantly affects gene expression in the epidermis through binding to the TCF bHLHs. TCF3/4/12 often heterodimerize with cell type specific class II bHLH factors (Murre et al., 1989a,b), thereby acquiring cell state and context specific target gene profiles. Expression and purification of TCF3 and TCF4 in primary epidermal progenitor cells, however, failed to identify additional bHLH interactors but could independently confirm the binding of TCF3 and TCF4 to ID1, respectively (Table S3). Collectively, these data suggest that ID1 binding to TCF3, TCF4 and TCF12 acts to modulate bHLH transcriptional programs during epidermal development.

Transcription factor binding motif analysis on promoter sequences in genes differentially expressed in *shId1* and ID1 overexpression (Fig. 5D, Q1 and Q3) using HOMER motif discovery revealed enrichment of bHLH motifs in potential ID1 targets when compared with all genes expressed in epidermal progenitors (Fig. 5F,G). Interestingly, motif discovery in genes significantly altered, but uncorrelated to ID1 levels (Fig. 5D, Q2 and Q4), revealed distinct motif profiles highlighting known regulators of epidermal differentiation such as grainyhead-like (GRHL), basic leucine zipper domain (bZIP), T-box and Trp63 transcription factors (Fig. 5F,G; Fig. S5G). Taken together, these data suggest that bHLH transcriptional programs impact epidermal progenitor states through modulation of specific target genes.

### CEBPA is ectopically expressed in epidermal progenitor cells in the absence of ID1

Having identified an *in vitro* ID1 gene signature harboring enrichment of bHLH binding motifs, we singled out CEBPA for further in-depth characterization. CEBPA is an interesting ID1 signature gene – the combined loss of CEBPA and CEBPB in the developing epidermis results in hyperproliferation and epidermal barrier defects (Lopez et al., 2009). We found prominent CEBPA expression in suprabasal layers at E14.5 and E16.5. In addition, a significant subset of K5-positive basal progenitors expressed lower levels of CEBPA (Fig. 5H). *In vitro* differentiation of epidermal progenitor cells mimicked the *in vivo* expression pattern with significant upregulation of *Cebpa* mRNA and protein concomitant with differentiation (Fig. S5H,I). In line with previous reports (Lopez et al., 2009), we found that doxycycline-mediated overexpression of CEBPA in cultured epidermal progenitors significantly reduced the number of EdU^+^ cycling progenitor cells (Fig. 5I,J; Fig. S5J). Quantification of EdU-incorporation in the K5-positive basal layer at E16.5 reveals that most proliferating cells are CEBPA negative (Fig. 5K,L), confirming a role for CEBPA in repressing epidermal progenitor cell proliferation. Supporting our *in vitro* sequencing data, shRNA silencing of *Id1* resulted in an increase in the number of K14/CEBPA double-positive progenitor cells compared with *shScr* at E16.5 (Fig. 5M,N), indicating that CEBPA expression is repressed in the presence of ID1 *in vivo*. To mechanistically explore whether *Cebpa* is transcriptionally regulated through an ID1-TCF axis, we cloned a 2 kb fragment of the *Cebpa* promoter as well as a *Cebpa* enhancer (Cooper et al., 2015) containing reported functional bHLH binding E-box motifs (Cooper et al., 2015; Hu et al., 2016; Pfurr et al., 2017; Soleimani et al., 2012; Yoon et al., 2015). We found an increase in promoter and enhancer luciferase reporter activity in *shId1* epidermal progenitor cells compared with *shScr* (Fig. S5K,L), correlating to the observed increase in mRNA and *in vivo* protein expression, suggesting that *Cebpa* regulatory elements are differentially engaged in the presence or absence of ID1.

### TCF3/4/12 localize to the developing epidermis and regulate progenitor cell proliferation

**Fig. 6.**
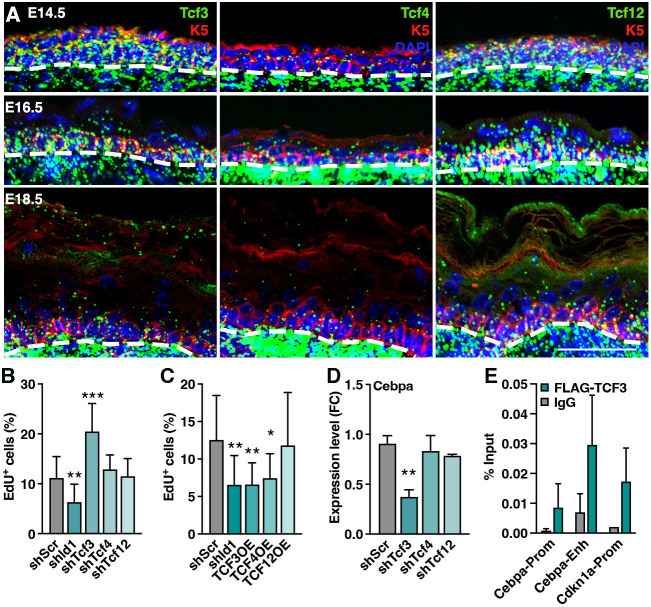
**TCF3/4/12 localize to the developing epidermis and regulate progenitor cell proliferation.** (A) Visualization of *Tcf3*, *Tcf4* and *Tcf12* mRNA (green) in skin reveal expression in epidermal progenitors at E14.5 and E16.5, with subsequent enrichment in basal layer upon induction of differentiation (E18.5). Dashed line indicates the boundary between the basal layer of the epidermis and dermis. (B) Silencing of *Tcf3*, but not *Tcf4* or *Tcf12*, promotes progenitor cell proliferation, *n*=12. (C) Overexpression of TCF3 and TCF4, but not TCF12, reduces proliferation *in vitro*, *n*=12. (D) Silencing of *Tcf3*, but not *Tcf4* or *Tcf12*, reduces *Cebpa* mRNA levels, *n*=3. (E) ChIP-qPCR for FLAG-TCF3 compared with IgG control demonstrated enrichment of TCF3 at the *Cebpa* promoter (Prom) and enhancer (Enh). *Cdkn1a* is used as positive control, *n*=3. Data are represented as mean±s.d. *P<0.05, **P<0.01, ***P<0.001 using multiple unpaired *t*-test. Scale bar: 50 μm.

TCF3, TCF4 and TCF12 are established class I bHLH ID interactors (Lasorella et al., 2001), but the expression dynamics and function of TCFs in the developing epidermis have not been elucidated. Returning to the E13 epidermal single-cell profiling (Fan et al., 2018), we found all three Tcf genes to be ubiquitously expressed in a majority of epidermal progenitor cells in both cluster 1 and 2, contrasting the cluster 1 enrichment of *Id1* (Fig. S6A,B). *In situ* hybridization confirmed the expression of *Tcf3*, *Tcf4* and *Tcf12* mRNA in K5-positive epidermal progenitors at E14.5 (Fig. 6A). Later in epidermal development (E16.5), *Tcf3*, *Tcf4* and *Tcf12* mRNA reactivity was apparent in both K5-positive progenitors as well as in suprabasal keratinocytes, only to become enriched in the K5 basal layer at E18.5 (Fig. 6A). We found decreasing expression of all Tcf genes during epidermal development (E14.5-E18.5) and higher epidermal mRNA reactivity for *Tcf3* and *Tcf12* compared with *Tcf4*. We also confirmed high expression of all three Tcf genes in the dermis at the time points analyzed (Rezza et al., 2016; Sennett et al., 2015). *In vitro* differentiation of cultured epidermal progenitors did not significantly alter the mRNA expression of *Tcf3*, *Tcf4* or *Tcf12* (Fig. S6C). Our data support a model in which the bHLH transcriptional output is controlled by spatially restricting ID protein expression.

To address the function of bHLH TCF transcription effectors in epidermal progenitor cells, we silenced *Tcf3*, *Tcf4* and *Tcf12* and assessed proliferation. Knockdown of *Tcf3*, but not *Tcf4* or *Tcf12*, resulted in increased EdU incorporation *in vitro* (Fig. 6B; Fig. S6D,F). In contrast, overexpression of TCF3 and TCF4, but not TCF12, reduced proliferation in cultured epidermal progenitor cells (Fig. 6C; Fig. S6E,G), suggesting that ID1 regulates epidermal progenitor cell proliferation through TCF3 (and possibly TCF4) -dependent transcriptional programs.

### *Cebpa* expression is bHLH-independent in epidermal progenitors

Our data suggest that activation of CEBPA is linked to silencing of *Id1*, *in vitro* and *in vivo*. To address whether ID1 acts to suppress *Cebpa* gene expression through sequestering of bHLH TCF transcriptional effectors, we assessed *Cebpa* mRNA expression after silencing or overexpression of TCF3, TCF4 and TCF12. Whereas silencing of *Tcf3* reduced *Cebpa* levels, overexpression of TCFs did not change *Cebpa* mRNA expression (Fig. 6D; Fig. S6H). As downregulation of TCF3 also positively affects progenitor proliferation, we performed chromatin immunoprecipitation (ChIP) followed by qPCR to determine a direct TCF3-*Cebpa* interaction. Using TCF3 binding to the *Cdkn1a* promoter as a positive control (Calero-Nieto et al., 2014; Hu et al., 2016; Pfurr et al., 2017; Wohner et al., 2016) we confirmed TCF3 binding to *Cebpa* promoter and enhancer chromatin (Fig. 6E). Modulation of TCF3 expression did not, however, correlate to alterations in *Cebpa* promoter or enhancer luciferase reporter activity (Fig. S6I,J), arguing that even though TCF3 is able to bind *Cebpa*, enhancing TCF3 activity alone is not sufficient to drive *Cebpa* transcription.

As our data suggest that upregulation of the differentiation marker *Cebpa* in *shId1*-targeted epidermis could not be explained by TCF3 activity alone, we asked whether silencing of *Tcf3* affects other genes associated with differentiation. Interestingly, epidermal progenitor cells targeted with *shTcf3*, but not consistently *shTcf4* or *shTcf12*, downregulated markers associated with differentiation which were found upregulated in *shId1*-targeted progenitors (Fig. S6K). Taken together, these data suggest that silencing of *Tcf3* affects markers of epidermal differentiation in progenitor cells.

### pSMAD1/5 activation of the *Id1* promoter is CEBPA-dependent

**Fig. 7.**
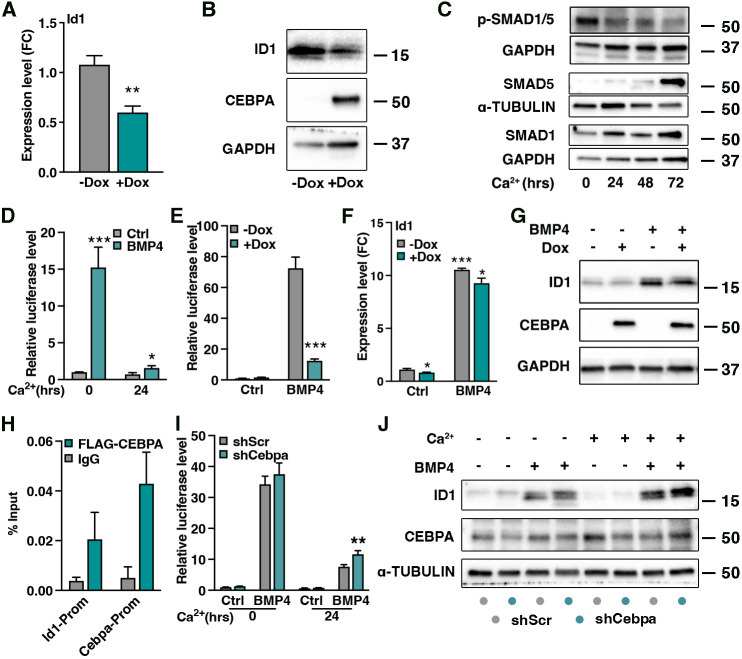
**pSMAD1/5 activation of the *Id1* promoter is CEBPA-dependent.** (A) Relative *Id1* mRNA levels upon 2 days of CEBPA overexpression (+Dox), *n*=3. (B) Protein level of ID1 is reduced upon forced CEBPA expression (+Dox). (C) pSMAD1/5 activity is pronounced in cultured epidermal progenitors and diminished upon differentiation. SMAD1 and SMAD5 are increased upon differentiation. (D) Luciferase reporter activity shows pronounced activity of a distal fragment of the *Id1* promoter in response to BMP in progenitors when compared to differentiated keratinocytes, *n*=3. (E) Forced CEBPA expression in epidermal progenitor cells inhibits the BMP-mediated activity of the *Id1* promoter, *n*=3. (F) Induction of *Id1* mRNA is impaired after BMP4 treatment in the presence of CEBPA (+Dox, green bar). Statistical comparisons are made between control groups (*), within −Dox conditions (gray bar to gray bar, ***) and between BMP4-treated groups (gray BMP4 to green BMP4, *), *n*=3. (G) Protein levels of ID1 are suppressed after BMP4 treatment when CEBPA levels are high. (H) ChIP-qPCR localizes FLAG-CEBPA at the *Id1* promoter. *Cebpa* promoter is used as positive control, *n*=3. (I) Statistical comparison made between *shScr* and *shCebpa* at 24 h of differentiation with BMP4, *n*=3. (J) Silencing of *Cebpa* enhances *Id1* promoter activity in differentiated keratinocytes and leads to upregulation of ID1. Data are represented as mean±s.d. *P<0.05, **P<0.01, ***P<0.001 using multiple unpaired *t*-test.

We noticed that modulating CEBPA expression affects *Id1* mRNA and protein (Fig. 7A,B; Fig. S7A), where high CEBPA expression acts to suppress ID1. *Id1* is a bona fide BMP target in the hair follicle stem cell lineage (Genander et al., 2014), and BMP signaling, as judged by phosphorylation of downstream effectors SMAD1/5, is active in epidermal progenitors and during *in vitro* differentiation (Fig. 7C; Fig. S7B-D). We assessed BMP sensitivity in epidermal progenitors and differentiated keratinocytes using a BMP-responsive pSMAD1/5 binding region located distally in the *Id1* promoter (Genander et al., 2014; Korchynskyi and ten Dijke, 2002). Whereas the pSMAD1/5 binding region in the *Id1* promoter responded to BMP in epidermal progenitor cells, keratinocytes differentiated for 24 h failed to efficiently activate *Id1* luciferase reporter activity in the presence of BMP (Fig. 7D), suggesting that pSMAD1/5 engagement with the *Id1* promoter is dynamically regulated during epidermal differentiation.

Chromatin binding of SMADs can be redirected upon expression of differentiation effectors (Trompouki et al., 2011). To this end, we overexpressed CEBPA in epidermal progenitor cells and found a dramatic reduction in BMP-induced response of the *Id1* promoter fragment (Fig. 7E), which correlated to reduced induction of *Id1* mRNA and protein (Fig. 7F,G). Comparing promoter activity and overall *Id1* mRNA/protein induction, the difference in reduction suggests that additional *Id1* promoter fragments drive *Id1* expression in the presence of CEBPA. ChIP-qPCR for FLAG-tagged CEBPA in epidermal progenitor cells unveiled binding of CEBPA to the BMP-responsive fragment of the *Id1* promoter [and *Cebpa* itself (Jakobsen et al., 2013; Meyer et al., 2016; Sun et al., 2018)], suggesting that CEBPA is able to modulate *Id1* transcriptional activity (Fig. 7H).

To ask if CEBPA normally acts to repress pSMAD1/5-dependent *Id1* expression, we silenced *Cebpa* in differentiated keratinocytes, a cell state in which CEBPA levels are endogenously high (Fig. 5). We found that downregulation of CEBPA potentiated BMP-mediated pSMAD1/5 activation of the *Id1* promoter in differentiated keratinocytes, albeit at a low level (Fig. 7I,J; Fig. S7E-G). Collectively, these results suggest that CEBPA can desensitize the *Id1* promoter elements to inductive BMP-mediated pSMAD1/5 transcription during epidermal commitment to differentiation.

## DISCUSSION

The transcriptional networks underlying progenitor differentiation in developing tissues are beginning to clear as more regulatory circuits are being identified. Here, we characterize transcriptional regulators in the embryonic skin to identify a new role for ID1 in epidermal development and place ID1 in a context of previously known epidermal effectors. We exploit developmental transitions forming the stratified epidermis and identify transcriptional crosstalk impacting progenitor cell states during epidermal development. Using published transcriptomics describing E13 epidermis (Fan et al., 2018), we identify *Id1* to be associated with progenitor cells committing to differentiation concurrent with stratification. Id genes have been previously implicated in human skin differentiation (Lopez-Pajares et al., 2015; Rotzer et al., 2006) and ID proteins are deregulated in human skin disease with impaired differentiation (Bjorntorp et al., 2003). Using *in utero* gene manipulation (Beronja et al., 2010), which, in contrast to transgenic recombination strategies (Andl et al., 2004; Vasioukhin et al., 1999), allows for targeting of the uncommitted single-layered epidermal progenitor population, we observe that ID1 promotes proliferative self-renewal (Fig. 4) and restricts commitment to differentiation during epidermal development *in vivo* (Fig. 3).

We find that progenitor cells devoid of ID1 are lost upon stratification (Fig. 2). Previous reports identified proliferation of basal progenitor cells as an inducer of epidermal stratification during development (Miroshnikova et al., 2018), where progenitor division results in local crowding, which induces differentiation and subsequent delamination. In contrast, stratification is suggested to precede progenitor proliferation in the adult epidermis (Mesa et al., 2018). Our work indicates that ID1-positive basal cells sustain progenitor states by coupling renewing proliferation with adhesion to the basement membrane (Figs 3 and 4). It is possible that *Id1*-silenced progenitors are outcompeted owing to additive defects in proliferation as well as adhesion. Although our *in vitro* transcriptional profiling cannot temporally delineate activation of differentiation markers from downregulation of anchoring proteins, our *in vivo* characterization indicates that delamination proceeds differentiation, identifying suprabasal K10-positive cells retaining cell cycle or basal K5 progenitor marker expression (Fig. 3). Interestingly, ID activity anchors neural stem cells to the ECM in the ventricular wall (Niola et al., 2012), suggesting that promotion of progenitor-to-niche adhesion is a common feature of ID-mediated transcriptional programs.

Although *Id1* is responding to canonical SMAD-mediated BMP signaling in cultured epidermal progenitor cells (Fig. 7), ID1-dependent phenotypes are not necessarily mirrored when altering upstream BMP signaling. Paradoxically, inhibition of BMP signaling through transgenic overexpression of BMP signaling inhibitors (He et al., 2002; Plikus et al., 2004; Sharov et al., 2003) results in epidermal hyperproliferation and reduced differentiation. In contrast, suprabasal BMP6 affects basal progenitor cell proliferation in a dose-dependent manner (Blessing et al., 1996), where low and high BMP6 induces and represses proliferation, respectively. Epidermal BMP signaling thus generally stimulates differentiation, while the effect on progenitor proliferation is dose- or context-dependent. To reconcile these phenotypes, BMP must, in addition to inducing *Id1* expression, exert ID1-independent effects on epidermal progenitor proliferation and differentiation, possibly through non-canonical BMP pathways (Botchkarev and Sharov, 2004).

ID1 promotes proliferation of immortalized keratinocytes *in vitro* (Rotzer et al., 2006) but antagonizes hair follicle stem cell (HFSC) activation *in vivo* (Genander et al., 2014). We establish ID1 as a positive regulator of epidermal proliferation *in vivo* (Fig. 4) and identify the interacting bHLH transcriptional factor TCF3 as a likely downstream effector. How ID1 promotes HFSC quiescence is unknown, but it is possible that HFSC-specific TCF heterodimerizing factors, lacking in the developing epidermis, direct the transcriptional response of TCF heterodimers in the absence of ID1. We were not able to identify additional heterodimerizing bHLH partners using cultured epidermal progenitors (Fig. 5), suggesting that TCF3 could regulate epidermal transcriptional targets as a TCF-TCF homodimer. Homodimerization of TCF3 has been demonstrated to control cell fate in pluripotent stem cells as well as in the B-cell and myogenic lineage (Neuhold and Wold, 1993; Rao et al., 2020; Shen and Kadesch, 1995). It would be interesting to functionally delineate the role of TCF dimerization in epidermal progenitors.

Focusing on CEBPA, an interesting regulator of cell cycle exit and lineage commitment (Lopez et al., 2009; Nerlov, 2007), we confirmed enrichment of CEBPA in *shId1*- compared with *shScr*-targeted progenitors *in vivo* (Fig. 5). Functional bHLH binding E-boxes are described in the *Cebpa* enhancer (Cooper et al., 2015) and promoter (Hu et al., 2016; Pfurr et al., 2017; Soleimani et al., 2012; Yoon et al., 2015) and, although we confirmed TCF3 binding to these chromatin regions (Fig. 6E), *Cebpa* luciferase reporter activity did not correlate to TCF3 levels. It is possible that overexpression of TCF3 induces a non-physiological response, explaining the lack of *Cebpa* activity, but we cannot exclude that ID1 acts upstream of *Cebpa* through TCF-independent mechanisms (Massari and Murre, 2000) and that CEBPA and TCF-mediated bHLH transcriptional programs act in parallel to cooperatively repress progenitor cell proliferation. However, it appears to be more likely, considering TCF3-binding to *Cebpa* chromatin, that additional so far unidentified co-factors are required to activate *Cebpa* transcription in a TCF-dependent manner, thereby acting to safeguard progenitor fate transitions towards differentiation.

We noticed that overexpression of CEBPA reduced *Id1* mRNA and protein levels, suggesting upstream regulation of *Id1* by CEBPA (Karaya et al., 2005; Mori et al., 2000; Saisanit and Sun, 1997). Exploiting a known BMP-sensing and pSMAD1/5-binding element in the *Id1* promoter (Genander et al., 2014; Korchynskyi and ten Dijke, 2002), we observed reduced BMP responsiveness upon differentiation or when CEBPA was introduced in progenitor cells and increased sensitivity in differentiated keratinocytes upon *Cebpa* silencing (Fig. 7). CEBPA can interact with SMAD4 (Coyle-Rink et al., 2002; Zauberman et al., 2001) and redirect chromatin binding of SMAD1 during lineage specification (Trompouki et al., 2011). We did not probe a direct CEBPA-SMAD interaction, but instead used ChIP to place CEBPA on the *Id1* promoter, suggesting that BMP and CEBPA act antagonistically to balance *Id1* transcription and thereby progenitor fate. Furthermore, the presence or absence of CEBPA determines the ability of pSMAD1/5 to activate BMP-sensing chromatin, potentially diversifying and balancing the transcriptional output in response to BMP and thereby fine-tuning epidermal lineage progression. More specifically, our work indicates that CEBPA-positive basal progenitors are refractory to BMP-induced *Id1* promoter activation and consequently fated towards epidermal differentiation. We identify ID1 as a new transcriptional effector coordinating epidermal development.

## MATERIALS AND METHODS

### Mouse husbandry

Animals were housed in pathogen-free conditions according to the recommendations of the Federation of European Laboratory Animal Science Association. All animal experiments were approved by Stockholms djurförsöksetiska nämnd (ethical permit no. N243/14, N116/16 and 14051-2019). *Id1* floxed mice have been previously described (Nam and Benezra, 2009), and Swiss mice were ordered (Janvier Labs) for *in utero* lentiviral injections with shRNAs.

### Genotyping

Ear or tail biopsies were lysed overnight at 55°C in DirectPCR lysis reagent (BioSite) in the presence of 0.1 mg/ml Proteinase K (Thermo Fisher Scientific). Lysis was stopped by heat inactivation (45 min, 85°C). Taq polymerase (5 U/µl, final concentration: 0.05 U/µl) was used for amplification in PCR buffer supplemented with 0.08 mM MgCl_2_, 0.2 mM dNTPs (all Invitrogen) and 0.4 µM primers (see Table S4). PCR products were analyzed with 2% agarose gel.

### Antibody staining

Tissue samples used in this study were either fixed before paraffin embedding or snap frozen and fixed before antibody staining with 4% formaldehyde (Sigma-Aldrich). Cells cultivated in chamber slides were subjected to the same fixative. For immunohistochemistry, antigen retrieval (R&D Systems) was performed, then blocking (Bloxall and Vector stain kit) and finally antibody incubation as indicated by the respective manufacturer's instructions. Samples used for immunofluorescence staining were cut at a thickness of 10 µm, blocked with goat serum (2.5%) and bovine serum albumin (1%) and permeabilized with 0.3% Triton X-100. When EdU was analyzed, the samples were treated with click chemistry before the antibody labeling (Click-iT, Thermo Fisher Scientific). Antibodies used for immunofluorescence staining were: ID1 (Biocheck, #BCH-1, 1:500), cleaved caspase-3 (Cell Signaling Technology, #9661, 1:400), K10 (Biolegend, #905404, 1:1000), K5 (Biolegend, #905901, 1:1000), K14 (OriGene, #BP5009, 1:1000), involucrin (Biolegend, #924401, 1:1000), TGM1 (Abcam, #ab103814, 1:1000), Flag (Sigma-Aldrich, F1804, 1:500), Cebpa (Cell Signaling Technology, #2295, 1:500). Antibodies used for immunoblotting were: Gapdh (Abcam, #ab8245, 1:5000), actin (Sigma-Aldrich, #A2228, 1:4000), tubulin (Abcam, #ab7291, 1:5000), ID1 (Biocheck, #BCH-1, 1:1000), Flag (Sigma-Aldrich, F1804, 1:5000), Cebpa (Cell Signaling Technology, #2295, 1:1000), phospho-Smad1/5 (Cell Signaling Technology, #9516, 1:1000). Smad1 (Cell Signaling Technology, #6944, 1:1000), Smad 5 (Cell Signaling Technology, #12534, 1:1000), Krt 10 (Covance, 90546, 1:1000).

### Plasmid information

Lentiviral plasmids used for silencing of *Id1*, *Cebpa* or *Tcf3/4/12* were generated by ligating the annealed short hairpin oligos into the pLKO.1 puro (Addgene plasmid #8453) or H2BGFP (Addgene plasmid #25999) vector backbone. The short hairpin sequences are listed in Table S1. The lentiviral plasmid expressing CRE and H2BRFP was modified from Addgene plasmid #25997. Expression plasmids coding for FLAG-tagged TCF3, TCF4 and ID1/2/3 were purchased from GeneCopoeia and used for co-immunoprecipitation experiments. Plasmids for inducible overexpression of ID1 and CEBPA were generated by Genescript from the pCW57.1 backbone (Addgene plasmid #38240).

### RT-qPCR

Cells and tissue samples were collected in Trizol LS Reagent (Thermo Fisher Scientific). RNA was extracted using RNAeasy (Qiagen) or Direct-zol (Zymo), and treated with DNaseI (Qiagen). After the quality check, 100 ng of total RNA was used for cDNA synthesis using SuperScript IV Vilo (Thermo Fisher Scientific). RT-qPCR was run with selected primer pairs and SYBR Green on a ViiA 7 device or a 7500 fast system (both Applied Biosystems). HPRT was used as an internal control. Primer sequences are listed in Table S1.

### RNAscope

RNA *in situ* hybridization to probe *Tcf3*, *Tcf4* and *Tcf12* was conducted using a Multiplex Fluorescent V2 kit from Advanced Cell Diagnostics, according to the manufacturer's instructions. In brief, FFPE slides were baked at 60°C for 1 h, followed by deparaffinization with two washes of xylene (5 min each) and two washes of 100% ethanol (2 min each). The slides were then dried for 5 min at 60°C, followed by incubation with hydrogen peroxide for 10 min at room temperature. The samples were then treated with protease at 40°C in the oven for 30 min, followed by incubation with *Tcf3*, *Tcf4* and *Tcf12* probes for 2 h at 40°C in the oven. After amplification with AMP (Amplification Reagent) 1, AMP 2 and AMP 3, each channel was developed by incubating HRP, fluorophore and HRP blocker sequentially based on the channels of the probe that was incubated. The samples were counterstained with DAPI (or antibodies if there were other channels available). Images were taken using a Zeiss Axioplan microscope and analyzed with Zen blue software.

### Single-cell RNA-sequencing

The datasets (E13_WT: 929 cells; E15_WT: 633 cells) provided by Fan et al. (2018) were obtained from the GEO database (GSE102086), transformed into a matrix and used as an input for the R package Seurat (version 2.3.0). For the E13 dataset, cells with less than 500 genes, more than 8500 genes and more than 5% mitochondrial genes were excluded from further analyses. Seurat was used for clustering of the dataset. Count data was log-normalized with a scaling factor of 10,000 before identification of variable features. Variable features were identified using variance stabilizing transformation and the top 2000 features were selected. Data scaling regressed out unwanted variation due to library size or mitochondrial gene content. Features were selected using principal component analysis (PCA). Nearest neighbors were identified using the first 17 principal components and clusters identified using a resolution of 0.5. Using the FindAllMarkers function, keratinocytes were identified as being positive for *Krt5* and *Krt15* expression and negative for *Pdgfra*, *Vim* and *Cd31* (*Pecam1*) (154 cells at E13). Using the function subset, re-analysis of the cells identified as keratinocytes was performed (variance stabilized transformed selecting top 2000 features, re-scaled data, re-ran PCA, performed nearest neighbor identification with six dimensions and clustering with a resolution parameter of 0.3) to visualize the expression of features of interest.

### ChIP and ChIP-qPCR

ChIP from mouse epidermal progenitor cells overexpressing FLAG-tagged CEBPA or TCF3 was performed using the MAGnify chromatin immunoprecipitation system (Invitrogen) according to the manufacturer's instructions. In brief, cells were fixed with 1% formaldehyde at room temperature for 10 min. The cross-linking was stopped by incubation with glycine (125 mM) for 5 min at room temperature. The cells were washed with ice-cold PBS twice and lysed in lysis buffer supplemented with protease inhibitor (1×10^6^ cells/50 µl lysis buffer), followed by sonicating into 200-500 bp fragments using a Bioruptor (Diagenode). The fragments were next incubated with magnetic beads, which were previously coupled with anti-Flag (Sigma-Aldrich, F1804) or IgG isotype (provided by the ChIP kit) antibodies at 4°C overnight. After washes with IP buffer1/2, the chromatin (including the input) was reverse cross-linked by incubation in reverse cross-linking buffer supplemented with proteinase K at 55°C for 15 min. The samples were then collected and purified with DNA purification magnetic beads, followed by eluting with 100 µl of elution buffer. The ChIP-qPCR was run with selected primer pairs and SYBR Green on a ViiA 7 device, and the calculations were based on the input and IgG isotype controls. Primer sequences are listed in Table S1.

### *In vitro* establishment and expansion of primary epidermal progenitors

Primary epidermal progenitor cells were established from postnatal day (P) 0 pups and maintained according to published protocols (Nowak and Fuchs, 2009) using 3T3-J2 feeders and E-Low media [75% DMEM (Gibco, 21068028), 25% F12 (Gibco,21700026), supplemented with 15% chelated fetal bovine serum (FBS) (Hyclone, #SH30071.03), 0.45 µg/ml hydrocortisone (Merck, 386698-25MG), 0.1125 nM Cholera toxin (Sigma-Aldrich, D0564-1MG), 50 µg/mL transferrin (Sigma-Aldrich, T-2252), 50 µg/ml insulin (Sigma-Aldrich, I-5500), 0.02 nM 3T (3,3′,5-triiodo-L-thyronine, Sigma-Aldrich, T-2752), 1× penicillin-streptomycin (Gibco, 15140122), 8 mM L-glutamine (Gibco, 25030081) and 0.03% sodium bicarbonate (Gibco, 25080094)]. E-low media contained 63.53 µM calcium and *in vitro* differentiation media contained 1.5 mM calcium. Epidermal progenitor cells expressing *shId1*, *shCebpa*, *shTcf3/4/12*, control *shScr* or protein coding sequences for ID1 or CEBPA were generated by lentiviral infection followed by puromycin (1 µg/ml) selection. ID1 and CEBPA expression was induced by doxycycline (1 µg/ml) for 3 days using the pCW75.1 backbone (Addgene plasmid #38240). Primary epidermal progenitors were within 20 passages.

### Production of high-titer lentivirus

Lentivirus was produced in 293TN cells (System Bioscience, LV900A-1) expanded in DMEM with 10% FBS, as previously described (Beronja et al., 2010), using pMD2/pPAX packaging plasmids (pMD2.G, Addgene plasmid #12259; psPAX2, Addgene, #12260) and calcium and the pLKO.1 (Addgene plasmid #10878) backbone. Epidermal progenitors were infected by spinoculation (1100 ***g*** for 30 min at 37°C) in the presence of 40 µg/ml hexadimethrine bromide (Beronja et al., 2010).

### *In utero* lentiviral injections

High titer lentivirus was injected into the amniotic cavity of E9.5 embryos in accordance with previous publications (Beronja et al., 2010) and with the help of Infinigene (Karolinska Institutet).

### Immunoprecipitation and mass spectrometry

The protein of interest (ID1, ID2, ID3, TCF3 or TCF4) was overexpressed by transfecting FLAG-tagged constructs into mouse epidermal progenitor cells (GeneCopoeia). Transfected cells were enriched using either puromycin or hygromycin and the FLAG-tag was precipitated using Sepharose beads pre-incubated with anti-FLAG M2 (Sigma-Aldrich, A2220) or IgG antibody. After incubation, beads were washed in Tris-buffered saline (TBS) and the precipitated protein was eluted using excess amounts of FLAG peptide (Sigma-Aldrich, final concentration 200 µg/ml in TBS). Efficiency of precipitations was confirmed using silver stain (Thermo Fisher Scientific, #24612) and, if approved, the eluate was forwarded for mass spectrometry analyses to the Proteomics Core facility, Karolinska Institutet. Interacting proteins were scored as hits based on the lack of signal in the IgG control, coverage and overall protein score.

### Luciferase reporter assay

The pGL3 basic backbone (Promega) was linearized by enzyme digestion with NheI and XhoI. Then the Id1 promoter region (336 bp), previously described by Genander et al. (2014), was cloned into the pGL3 backbone with Infusion (Takara). Mouse *Cebpa* promoter (−2 kb) and enhancer regions (Cooper et al., 2015) were amplified using the following primer pairs: Cebpa_Prom_Infu_F TCTTACGCGTGCTAGCGCAGGGACATTTCTCACGAACC; Cebpa_Prom_Infu_R GATCGCAGATCTCGAGGAGTTAGAGTTCTCCCGGCA; Cebpa_Enh_Infu_F TATCGATAGGTACCGAGCTCCACCCCCTGATTTGCCATTCAT; Cebpa_Enh_Infu_R GCCTATCGAGCCCGGGTCCCAGGTCCCACCATACCTG.

The empty pGL3 backbone was used for background control. Epidermal progenitor cells were co-transfected with the luciferase construct and *Renilla*-luciferin 2-monooxygenase RLuc transfection baseline control (in a ratio of 1:10) using Lipofectamine LTX (Thermo Fisher Scientific). Lysates were measured for luciferase activity and RLuc using the Dual Glo luciferase assay system on a Glomax reader (both Promega). To assess BMP sensitivity, cells were serum starved overnight and treated with BMP4 (200 ng/ml) for 3 h before being lysed.

### RNA-sequencing and bioinformatic analyses

Total RNA was analyzed on a bioanalyzer and used for library preparation. Samples were sequenced using a NovaSeq6000 platform (Illumina). A quality check (Fast QC/0.11.5) was run on raw sequencing reads. Raw sequencing reads were processed to obtain counts per genes for each sample. The EdgeR package was used to normalize for the RNA composition by finding a set of scaling factors for the library sizes that minimize the log-fold changes between the samples for most genes, using a trimmed mean of M values (TMM) between each pair of samples. Homer/4.10 was used to analyze promoters of genes and look for motifs that were enriched in the target gene promoters relative to all other promoters in the genome. Analyses, statistical computing and graphics were performed using R and in collaboration with National Bioinformatics Infrastructure Sweden (NBIS) at Karolinska Institutet.

### Statistical analyses

GraphPad Prism and R were used for statistical analyses, including the use of multiple unpaired *t*-test and ANOVA when applicable. Error bars show the standard deviation or standard error of the mean as specified in the figure legends. Gene enrichment was analyzed using Gene Ontology (Ashburner et al., 2000). The *n* of *in vivo* quantifications is specified in the corresponding figure legend. *In vitro* experiments have been performed multiple times to verify the conclusions displayed in the representative figure.

## Supplementary Material

10.1242/develop.201262_sup1 Supplementary information
