# Supplementary material for: ID1 and CEBPA coordinate epidermal progenitor cell differentiation
Source: Development. 2022 Nov 16;149(22):dev201262. doi: 10.1242/dev.201262 (PMC9845743; doi:10.1242/dev.201262)
Supplement: Supplementary information [file develop-149-201262-s1.pdf]

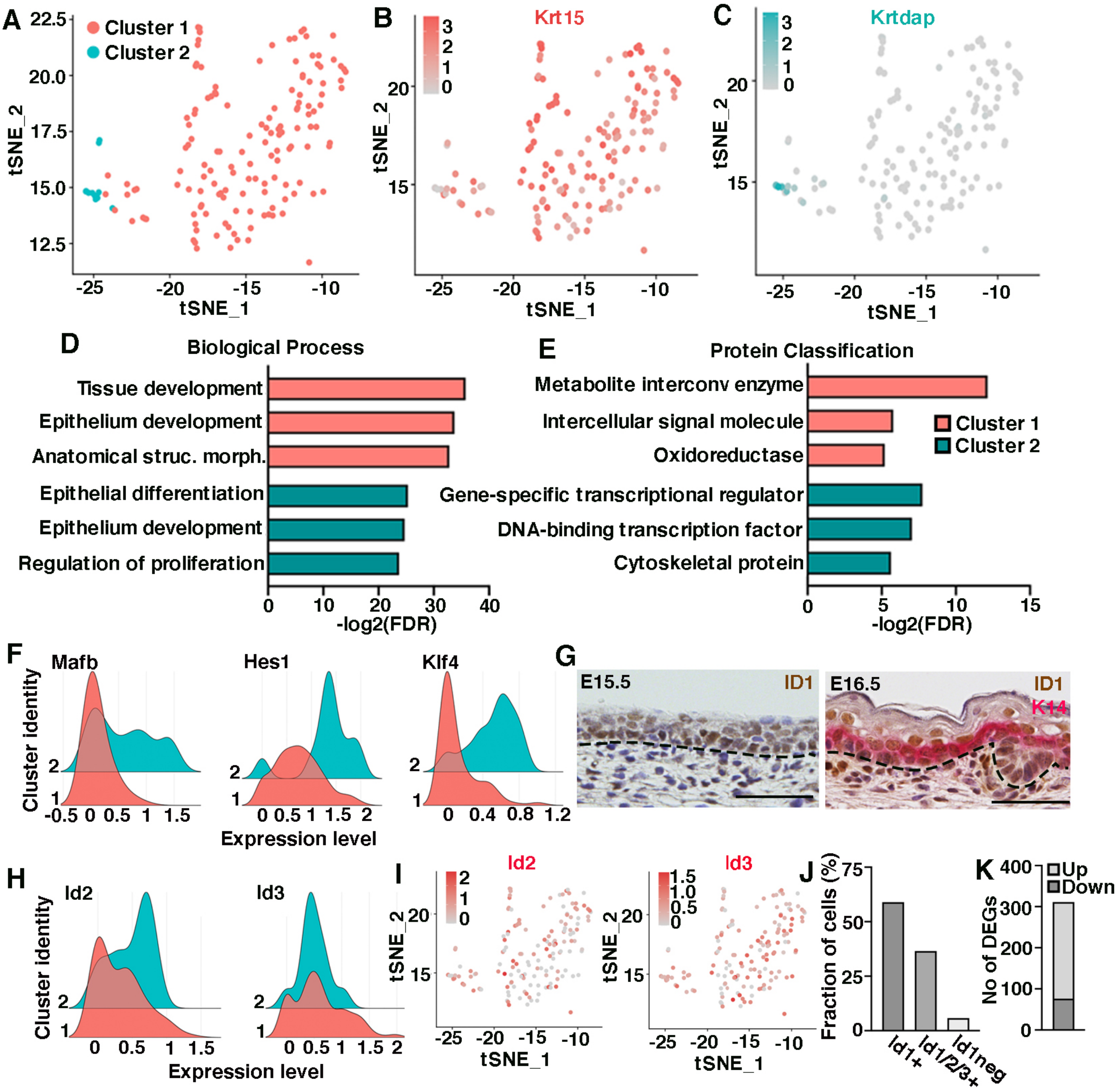

Fig. S1. ID1 is expressed in epidermal progenitor cells during skin development

(A) Feature plot displaying cluster 1 and cluster 2 from E13 epidermal single-cell RNA-sequencing.

(B and C) Basal marker *Krt15* is enriched is cluster 1, whereas *Krtdap*, a marker of epidermal differentiation, is exclusively expressed in cluster 2.

(D) Gene Ontology analysis of biological processes using genes enriched in cluster 1 or 2.

(E) Protein classification of genes enriched in cluster 1 and cluster 2 respectively.

Development • Supplementary information

(F) Ridge plots of know markers of epidermal differentiation (*Mafb*, *Hes1* and *Klf4*) enriched in cluster 2.

(G) ID1 protein expression in developing epidermis at E15.5 and E16.5.

(H and I) Ridge and feature plots of *Id2* and *Id3* expression in E13 epidermis.

(J) Percentage of sequenced E13 epidermal progenitors expressing *Id1* alone, *Id1/2/3* together or are *Id1* negative.

(K) Number of differentially expressed genes found in *shId1* targeted cultured epidermal progenitors when compared to *shScr* cells.

Scale bars 75 µm (G, E15.5) 50 µm (G, E16.5).

Development • Supplementary information

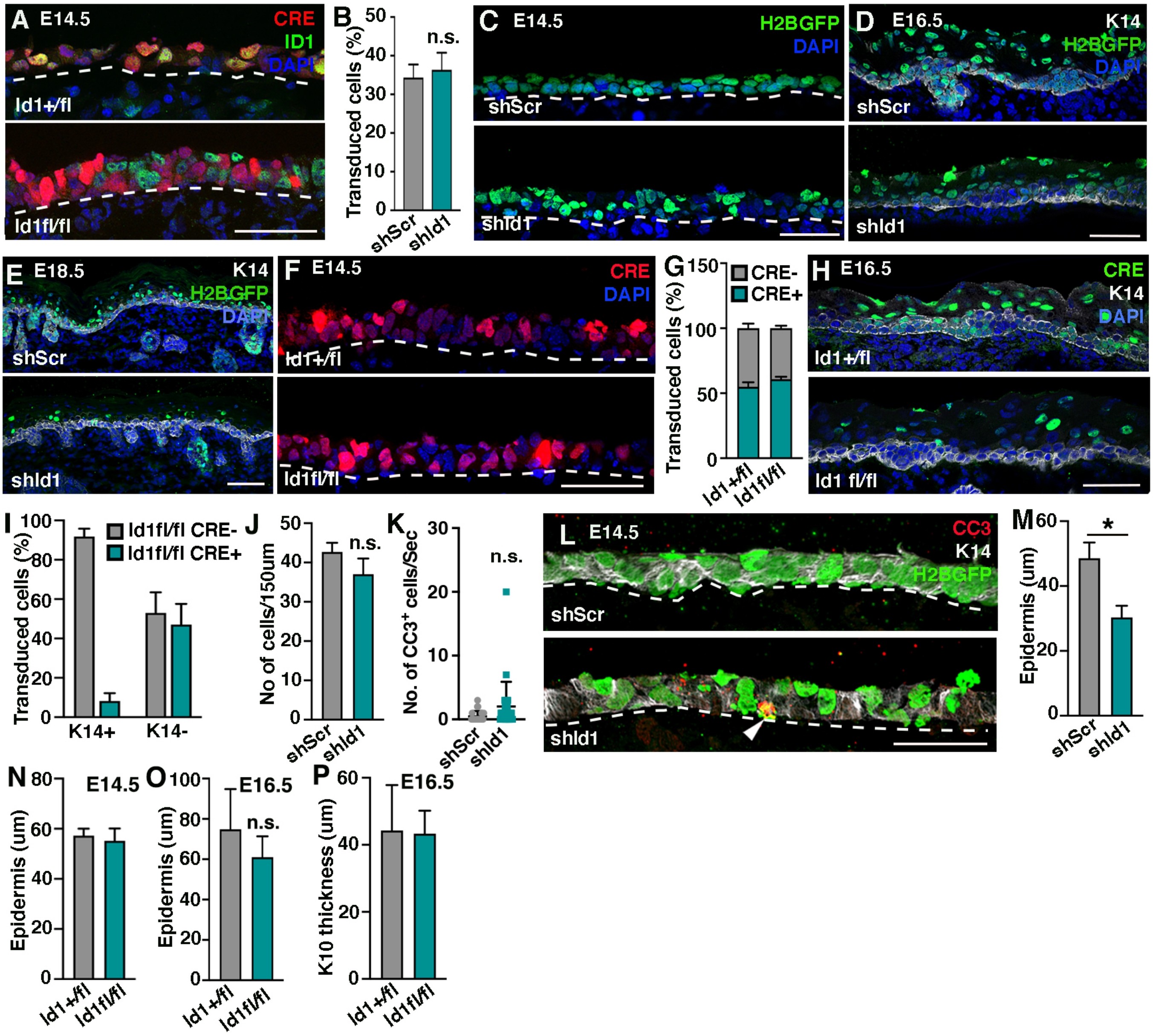

Fig. S2. ID1 counteracts epidermal progenitor delamination

(A) ID1 immunoreactivity is reduced in *Id1*$^{fl/fl}$ epidermis, but not *Id1*$^{+/fl}$, targeted with LV-CRE.

(B) *In vitro* transduction efficiency of *shScr* and *shId1* are comparable.

(C-E) Localization of transduced H2BGFP reporter positive cells at E14.5, E16.5 and E18.5 in *shScr* and *shId1* targeted epidermis.

(F and G) Distribution and quantification of LV-CRE targeted cells in *Id1*$^{+/fl}$ and *Id1*$^{fl/fl}$ at E14.5.

(H and I) Distribution and quantification of LV-CRE targeted K14-positive and K14-negative cells in $Id1^{+/fl}$ and $Id1^{fl/fl}$ at E16.5.

(J) Quantification of the number of cells/150um at E14.5 in *shScr* compared to *shId1* targeted epidermis.

(K and L) Immunoreactivity against cleaved caspase-3 (CC3) is not altered upon *Id1* silencing at E14.5. Embryos, shScr: n=4, shID1: n=5, sections for each embryo: n=3.

(M) Measurement of epidermal thickness at E16.5 in *shScr* and *shId1*.

(N and O) Thickness of epidermis in $Id1^{+/fl}$ and $Id1^{fl/fl}$ targeted epidermis at E14.5 and E16.5.

(P) K10 spinous layer thickness in in $Id1^{+/fl}$ and $^{Id1fl/fl}$ targeted epidermis at E16.5.

Data are represented as mean ± SEM. $^{*}p < 0.05$ using unpaired t-test. Scale bars 50 µm. n=3-6 in all quantifications (G, I, J, K, N-P), n=2 and 6 in M.

Development • Supplementary information

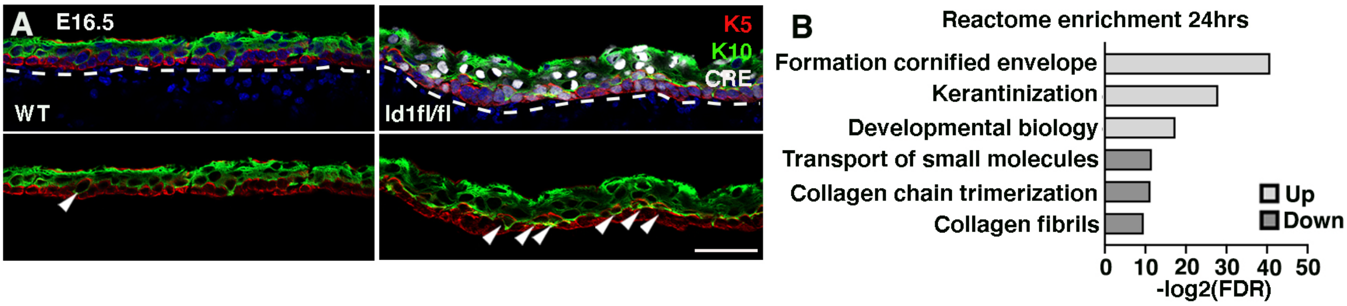

Fig. S3. Progenitor cells devoid of ID1 co-express basal and differentiation markers

(A) Immunoreactivity for K5 and K10 shows increased number of double-positive cells in *Id1*$^{fl/fl}$ skin targeted with LV-Cre compared to wild type epidermis.

(B) Reactome enrichment for genes differentially expressed at 24 hours of differentiation in *shId1* targeted cultured epidermal progenitors compared to *shScr* cells (>2xFC).

Scale bars 50 µm.

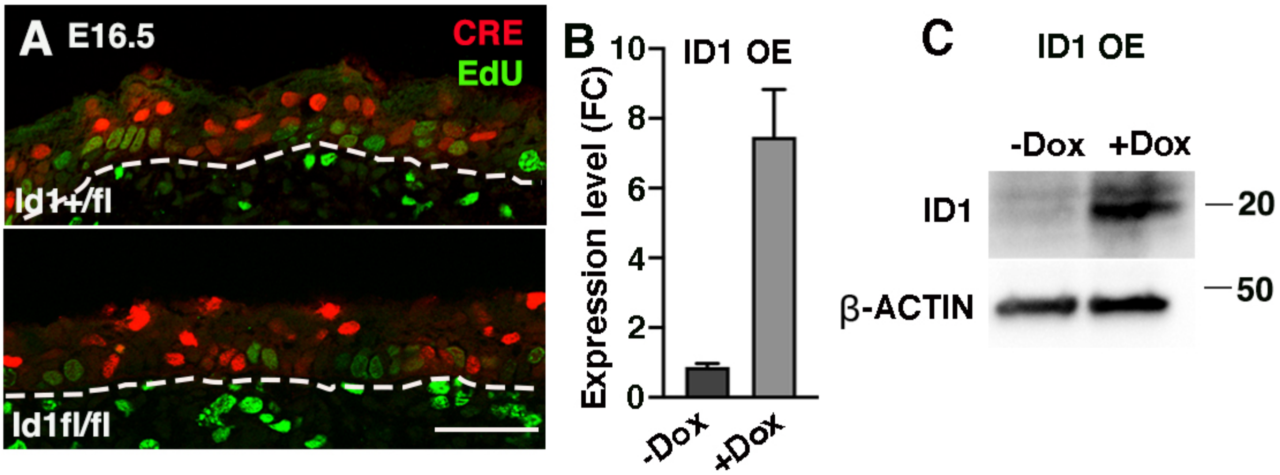

Fig. S4. Epidermal progenitor proliferation is positively regulated by ID1

(A) Combined CRE and EdU immunoreactivity in *Id1*$^{+/fl}$ compared to *Id1*$^{fl/fl}$ epidermis at E16.5.

(B) *Id1* mRNA levels after Dox induction in cultured epidermal progenitors.

(C) ID1 protein is enriched in epidermal progenitors in doxycycline (Dox) treated cells compared to untreated cultures.

Scale bars 50 µm.

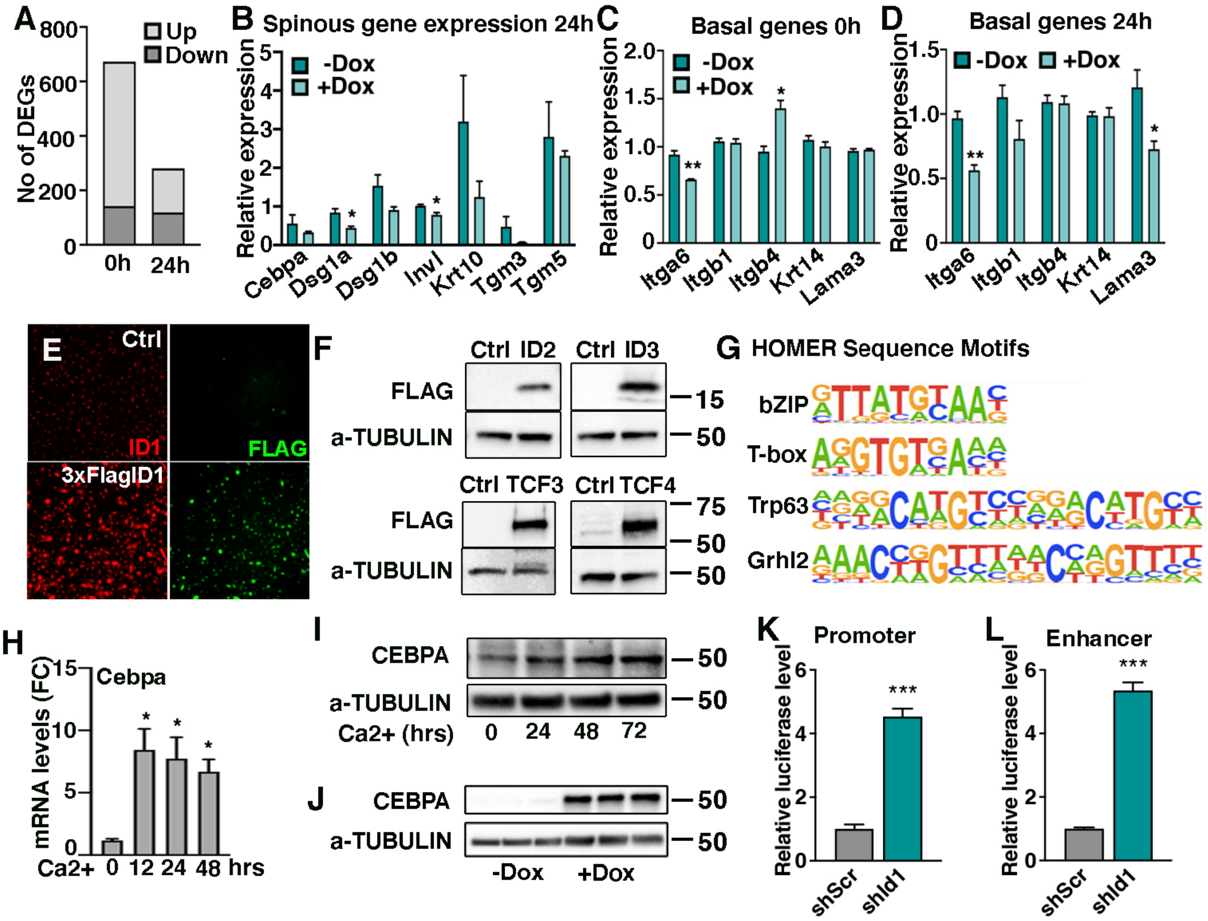

Fig. S5. Identification of ID1 gene signatures

(A) Number of differentially expressed genes in ID1 overexpressing epidermal progenitor cells (0 hours) asked to differentiation (24 hours).

(B) Spinous markers expression is impaired at 24 hours of differentiation in ID1 overexpressing epidermal cells.

(C and D) Basal gene markers are not affected by ID1 overexpression.

(E and F) Overexpression of FLAG-tagged ID1, ID2, ID3 TCF3 and TCF4 in cultured epidermal progenitors.

(G) HOMER motif analysis reveals enrichment of bZIP (basic leucine zipper domain), T-box, Trp63 and GRHL sequence motifs in promoters (+400bp) of Q2+Q4 genes when compared to all other expressed gene promoters.

Development • Supplementary information

(H) *Cebpa* mRNA levels increase with differentiation of epidermal progenitor cells. One way ANOVA comparing 24, 48 and 72 hours to 0 hours. n=3.

(I) CEBPA protein levels are increased following *in vitro* differentiation of epidermal progenitors.

(J) Doxycycline dependent (2 days treatment) overexpression of CEBPA in epidermal progenitor cells.

(K and L) *Cebpa* promoter and enhancer luciferase reporter activity in s *hScr* and *shId1* targeted progenitors, n=3. Data are represented as mean ± SEM. *$p < 0.05$, ***$p < 0.001$ using multiple unpaired t-test and ANOVA.

Development • Supplementary information

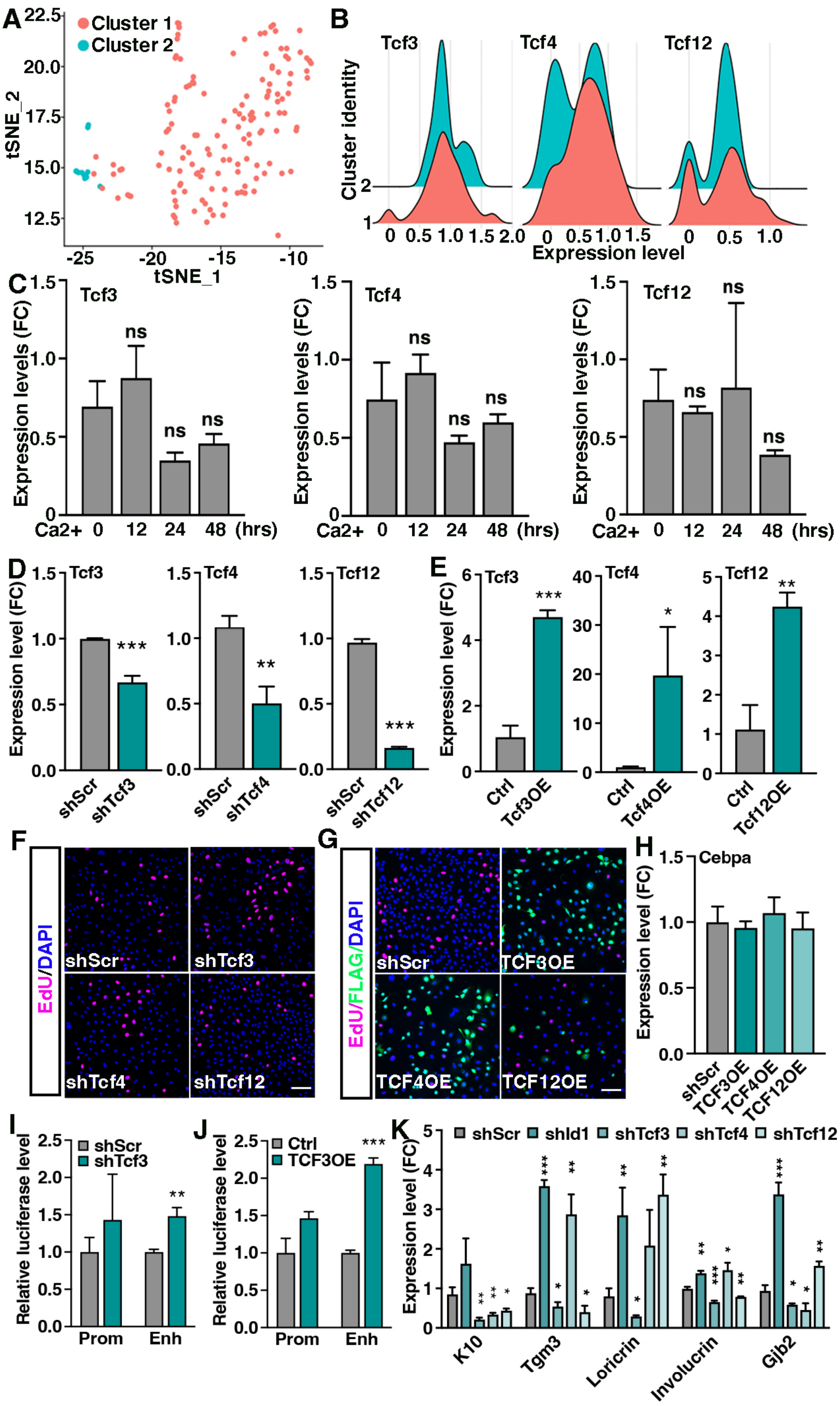

Development • Supplementary information

Fig. S6. TCF3/4/12 localize to the developing epidermis and regulate progenitor cell proliferation

(A and B) *Tcf3*, *Tcf4* and *Tcf12* are uniformly expressed in both cluster 1 and 2 in E13 epidermis.

(C) Relative mRNA expression levels of *Tcf3*, *Tcf4* and *Tcf12* upon *in vitro* differentiation of epidermal progenitor cells. Statistical analysis using ANOVA fails to detect significant alterations of *Tcf* expression correlating to epidermal differentiation, comparing 24, 48 and 72 hours to 0 hours.

(D) Knock down efficiency in epidermal progenitor cells *in vitro* using shRNAs targeting *Tcf3*, *Tcf4* or *Tcf12*, n=3.

(E) Overexpression efficiency (fold change mRNA) of TCF3, TCF4 and TCF12 in epidermal progenitor cells, n=3.

(F) Representative images showing EdU incorporation in keratinocytes targeted with *shScr, shTcf3, shTcf4,* and *shTcf12*.

(G) Representative images showing EdU incorporation in keratinocytes overexpressing TCF3, TCF4, and TCF12 (transient overexpression in *shScr* keratinocytes).

(H) *Cebpa* mRNA is not altered upon forced single TCF expression, n=3.

(I-J) *Cebpa* promoter (+2kb fragment) and enhancer (Cooper *et al*., 2015) luciferase activity upon *Tcf3* silencing (I) and overexpression (J), n=3.

(K) Expression profiles of differentiation markers in epidermal progenitor cells after silencing of *Id1*, *Tcf3*, *Tcf4* or *Tcf12*, n=3.
Data are represented as mean ± SD. *p < 0.05 **p < 0.01 ***p < 0.001 using multiple unpaired t-test. Scale bar 100 μm.

Development • Supplementary information

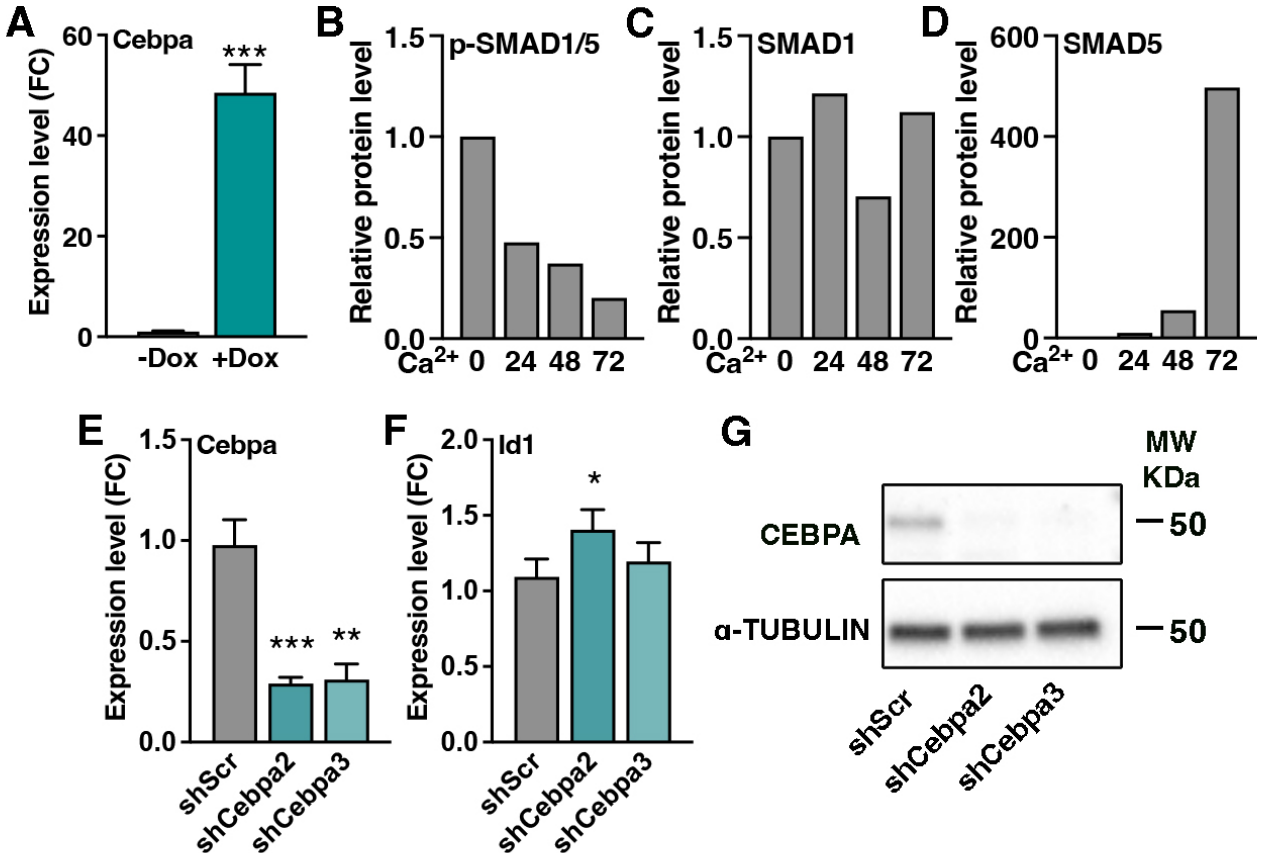

Fig. S7. pSMAD1/5 activation of the *Id1* promoter is CEBPA dependent

(A) mRNA induction of *Cebpa* upon doxycycline treatment, n=3.

(B-D) Quantification of protein levels p-SMAD1/5, SMAD1 and SMAD5 compared to loading controls (see main Fig. 7C).

(E)Relative silencing of *Cebpa* mRNA using two different shRNAs, n=3.

(F) *Id1* mRNA levels after silencing of *Cebpa* in epidermal progenitors, n=3.

(G) Protein levels of CEBPA after shRNA silencing in progenitor cells.

Data are represented as mean ±SD. *$p < 0.05$ **$p < 0.01$ ***$p < 0.001$ using multiple unpaired t-test.

Development • Supplementary information

Table S1. Differentially expressed genes in E13 epidermis comparing cluster 1 to cluster 2.

Click here to download Table S1

Table S2. Gene list of differentially expressed genes from Figure 5D, Q1 and Q3.

Click here to download Table S2

Table S3. Mass spectrometry data from overexpression of co-immunoprecipitation of 3xFLAG-ID1, ID2, ID3, TCF3 and TCF4. Proteins listed were identified in duplicate experiments, and not in IgG control samples.

Click here to download Table S3

Table S4. Primer sequences

Click here to download Table S4

Development • Supplementary information
